# Maternal phthalate exposure and asthma, rhinitis and eczema in 552 children aged 5 years; a prospective cohort study

**DOI:** 10.1186/s12940-020-00586-x

**Published:** 2020-03-13

**Authors:** Camilla Jøhnk, Arne Høst, Steffen Husby, Greet Schoeters, Clara Amalie Gade Timmermann, Henriette Boye Kyhl, Iben Have Beck, Anna-Maria Andersson, Hanne Frederiksen, Tina Kold Jensen

**Affiliations:** 1 Department of Environmental Medicine, Institute of Public Health, University of Southern Denmark, J.B. Winsløwsvej 17A, 5000 Odense, Denmark; 2 Hans Christian Andersen Children’s Hospital, Odense University Hospital, Odense, Denmark; 3 Environmental Risk and Health Unit, Flemish Institute for Technological Research (VITO), Mol, Belgium; 4 Department of Biomedical Sciences, University of Antwerp, 2000 Antwerp, Belgium; 5 Odense Patient data Explorative Network (OPEN), Odense, Denmark; 6 Department of Growth and Reproduction, Rigshospitalet, Copenhagen University Hospital, Copenhagen, Denmark; 7 International Center for Research and Research Training in Endocrine Disruption of Male Reproduction and Child Health (EDMaRC), Rigshospitalet, University of Copenhagen, Copenhagen, Denmark

**Keywords:** Phthalate exposure, Endocrine disrupting chemicals, Asthma, Allergic rhinitis, Eczema

## Abstract

**Background:**

Prenatal phthalate exposure has been suggested to alter immune responses and increase the risk of asthma, eczema and rhinitis. However, few studies have examined the effects in prospective cohorts and only one examined rhinitis. We therefore studied associations between maternal urinary concentrations of phthalate metabolites and asthma, eczema and rhinitis in offspring aged 5 years.

**Methods:**

From 552 pregnant women in the Odense Child Cohort, we quantified urinary concentrations of 12 phthalate metabolites in third trimester. We assessed asthma, rhinitis and eczema in their offspring at age 5 years with a questionnaire based on the International Study of Asthma and Allergies in Childhood (ISAAC), and conducted logistic regression adjusting for relevant confounders.

**Results:**

7.4% of the children had asthma, 11.7% eczema and 9.2% rhinitis. Phthalate exposure was low compared to previous cohorts. No significant associations between prenatal phthalate exposure and asthma were found. Odds ratios (ORs) of child rhinitis with a doubling in ΣDiNP_m_ and di-2-ethylhexyl phthalate metabolite (ΣDEHP_m_) concentrations were, respectively, 1.15 (95% confidence interval (CI) 0.97,1.36) and 1.21 (CI 0.93,1.58). The OR of eczema when doubling ΣDiNP_m_ was 1.24 (CI 1.00,1.55), whereas the OR of using medicine against eczema when doubling a di-ethyl phthalate (DEP) metabolite was 0.81 (CI 0.68,0.96).

**Conclusion:**

The lack of association between maternal phthalate exposure and asthma in the offspring may be due to low exposure and difficulties in determining asthma in 5-year-olds. The higher odds of rhinitis may raise public concern but further research in larger cohorts of older children is warranted.

## Background

Asthma is the most common chronic condition among children with an estimated lifetime prevalence on 12% in Danish 5-year olds [1]. It is commonly seen in a triad with atopic dermatitis and allergic rhinitis. The lifetime prevalence [1] of atopic dermatitis at age 5 years in Danish children is 13%, and of rhinoconjunctivitis it is 7%. Asthma is characterized by recurrent airway obstruction and bronchial hyper-responsiveness [2]. In many asthmatic children, wheezing and respiratory symptoms can be traced back to early childhood [3]. The diagnosis in young children is symptom based, as they cannot collaborate in spirometry [2]. However, asthma symptoms resemble symptoms of common infections like bronchitis.

Phthalates are synthetic chemical compounds used as plasticizers in consumer products. They form weak chemical bonds and are easily leached into the surrounding environment, and they can cross the placenta and affect the fetus [4]. Phthalates have short biological half-lives from hours to a few days, but because exposure is ubiquitous, exposure levels may be relatively constant [5, 6]. We identified 14 prospective cohort studies [5, 7–19] examining the association between prenatal phthalate exposure and asthma or allergy. Findings are inconsistent probably because different metabolites have been measured and the age of the children varied. Three studies [9, 11, 16] measured metabolites of diisononyl phthalate (DiNP) and only one [13] studied rhinitis.

To our knowledge, no Scandinavian prospective cohort studies have examined the association between in utero phthalate exposure and development of asthma and allergy, and we therefore used data from a prospective Danish birth cohort to examine the association between maternal urinary phthalate metabolite concentrations in third trimester and development of asthma, eczema and rhinitis in 552 offspring aged 5 years.

## Materials and methods

### Study settings and participants

From 2010 to 2012 all newly pregnant women residing in Odense were invited to participate in the Odense Child Cohort (OCC) at Odense University Hospital between gestational age (GA) 10–16 weeks (earlier described in detail [20]). Information on maternal pre-pregnancy Body Mass Index (BMI), maternal educational level and maternal smoking during pregnancy was obtained through questionnaires during pregnancy. Data on birth characteristics, maternal age, parity at inclusion and GA was obtained from hospital records. Preterm was defined as born before GA 37 weeks. Information on breastfeeding, smoking, pets and family history of asthma and allergy was obtained from questionnaires during the first 5 life years.

### Phthalate metabolite measurements

At approximately GA 28 weeks 870 pregnant women delivered a fasting spot urine sample for analysis of total content (free and conjugated) of 12 phthalate metabolites, of which some were summed (previously published [21]). The 4 di-2-ethylhexyl phthalate (DEHP) metabolites mono-2-ethylhexyl phthalate (MEHP), mono-2-ethyl-5-hydroxyhexyl phthalate (MEHHP), mono-2-ethyl-5-oxohexyl phthalate (MEOHP), mono-2-ethyl-5-carboxypentyl phthalate (MECPP) where measured and summed as ΣDEHP_m_ by addition of the molar sum of the four metabolites multiplied by the molecular weight of DEHP. Likewise, the 4 measured DiNP metabolites; mono-iso-nonyl phthalate (MiNP), mono-hydroxy-iso-nonyl phthalate (MHiNP), mono-oxo-iso-nonyl phthalate (MOiNP) and mono-carboxy-iso-octyl phthalate (MCiOP) were measured and summed as ΣDiNP_m_. We measured mono-iso-butyl phthalate (MiBP) and mono-*n*-butyl phthalate (MnBP) and combined them as MBP_i + n_, since the two metabolites have been shown to be highly correlated in the urine of the women of Odense Child Cohort [22, 23]. Finally, we measured mono-benzyl phthalate (MBzP) and monoethyl phthalate (MEP). Moderate to high correlation between all summed metabolites has been shown elsewhere [23]. Preparation and storage of standard solutions and samples, instrumental LC-MS/MS analysis and general method validation has previously been described [22].

To correct for urinary dilution, all samples with a phthalate metabolite concentration above the limit of detection (LOD) were osmolality adjusted. Osmolality was measured by the freezing point depression method [24]. All measured urinary concentrations were subsequently normalized to the group median osmolality (0.63 x osm/kg) using the following equation:

$$ \mathrm{Osmolality}\ \mathrm{adj}.\mathrm{conc}.\left(\mathrm{ng}/\mathrm{mL}\right)=\frac{\mathrm{urinary}\ \mathrm{conc}.\left(\mathrm{ng}/\mathrm{mL}\right)\times 0.63\ \left(\mathrm{osm}/\mathrm{kg}\right)}{\mathrm{sample}\ \mathrm{osmolality}\ \left(\mathrm{osm}/\mathrm{kg}\right)} $$

This adjustment method was chosen because osmolality is less affected by other exogenous factors than other adjustment methods [25]. Concentrations below the LOD were substituted by the metabolite specific LOD divided by the square root of 2.

### Measurement of asthma and allergy

A Danish modified version of The International Study of Asthma and Allergies in Childhood (ISAAC) was administered at the 5 years’ examination. Asthma outcomes were wheeze within the last 2 years, self-reported asthma, doctor diagnosed asthma and use of medicine to treat asthma/cold within the last 12 months. Self-reported asthma was defined as at least 3 episodes of wheeze (each lasting more than a day) within the last year. We used three different eczema outcomes; self-reported eczema, doctor-diagnosed eczema and use of prescribed medicine against atopic dermatitis or eczema of flexural folds. Self-reported eczema was defined as itchy symmetric eczema in the flexural folds behind the knees (which possibly came and disappeared again) within the last 6 months. Doctor diagnosed and use of prescribed medicine encountered all five life years, not just the last 6 months. Finally, we studied self-reported and doctor-diagnosed rhinitis defined as problems with recurrent sneezing and/or runny nose without having a cold or the flu and ever doctor diagnosed with hay fever. Due to a very low prevalence (1.3%) of doctor-diagnosed rhinitis, we did not study this outcome. Doctor diagnosed outcomes were all parental report of doctor’s diagnosis.

### Data analysis

The children included in this study were first compared to the rest of the OCC population with regard to child- and upbringing characteristics using t-tests and chi2-tests for continuous and categorical variables, respectively. We then compared characteristics among participating children with and without asthma, eczema or rhinitis using the same tests. Median and 25–75 percentiles of phthalate metabolite concentrations were compared according to the mother-child and upbringing characteristics and differences in the distribution assessed by Mann Whitney and Kruskal-Wallis test. Furthermore, Kruskal Wallis test was used to test for differences in maternal phthalate metabolite concentrations between children with two, one and no allergic outcomes.

**Fig. 1:**
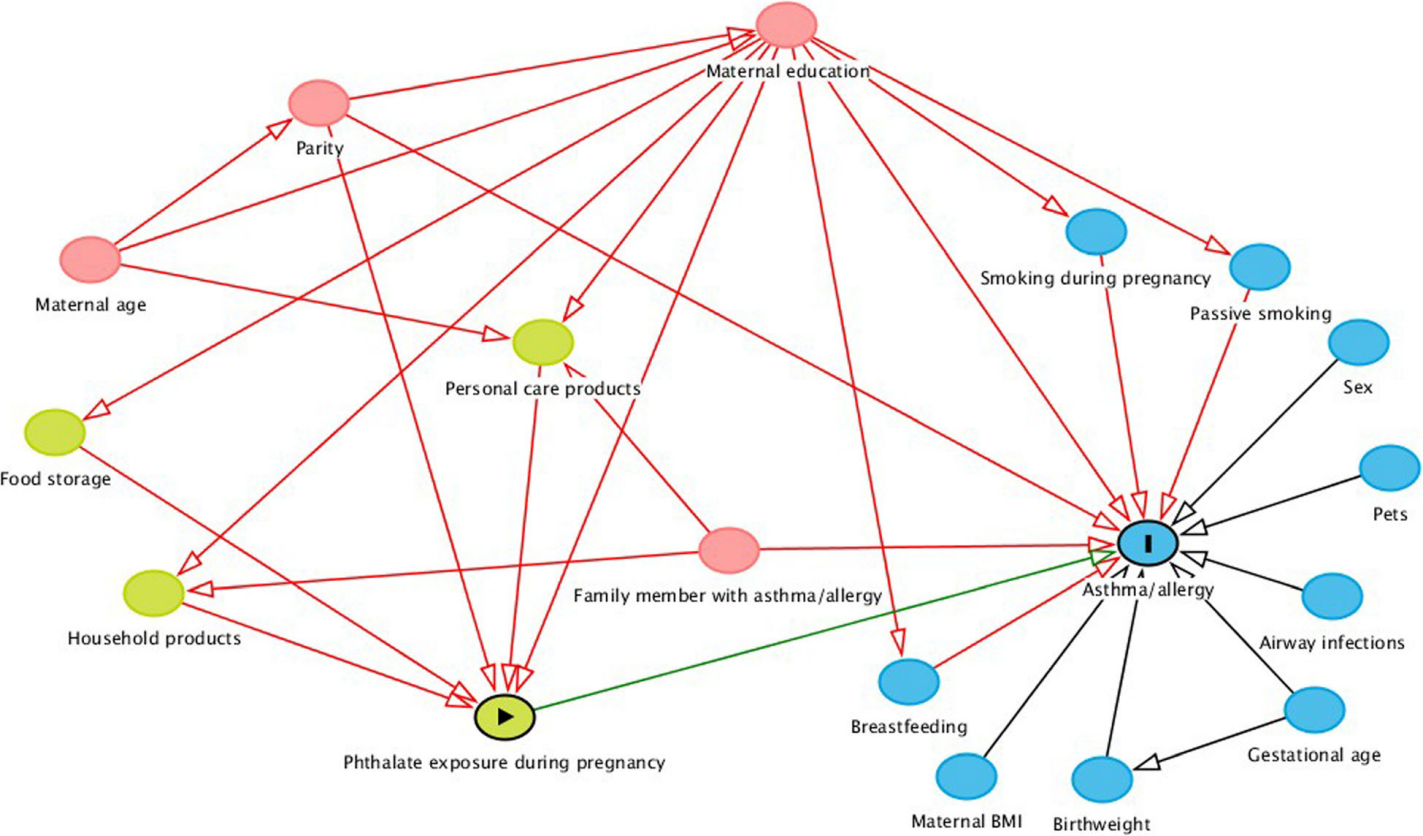
Directed acyclic graph (DAG) for evaluation of covariates in the logistic regression model. Legends: DAG for evaluation of covariates in the association between phthalates and asthma/allergy. Green nodes: Variables associated with maternal phthalate exposure. Blue nodes: Variables associated with asthma in the offspring. Red nodes: Variables associated with phthalates and asthma outcomes

The distribution of phthalate metabolite concentrations was right-skewed and transformed by the natural logarithm (ln) to normalize distribution. The associations between ln transformed phthalate exposure and allergic outcomes were assessed using logistic regression. Phthalate metabolite concentrations were then back transformed to study the odds ratios (OR) for a doubling of phthalate exposure. We identified potential confounders using a directed acyclic graph based on existing literature (Fig. 1). Maternal age, educational level and parity were a priori expected to influence both exposure to phthalates and development of asthma, eczema and rhinitis and adjusted for in the final model to reduce model variance. Having a parent or sibling with allergy is a strong predictor for childhood allergy and was therefore included in the final model. In utero exposure to maternal smoking is a well-known risk factor for development of asthma, eczema and rhinitis, however, only 3% of mothers smoked during pregnancy, and this was therefore not included in the final model. To evaluate sex interactions, the interaction term of sex and phthalate metabolite were added in each model. However, the interaction was not significant, and was therefore removed from the final model.

Statistical analyses were conducted using STATA/IC 15.0, and *p*-values < 0.05 were considered significant.

## Results

Totally, 846 women had phthalate metabolites measured in their urine and 1316 parents of singleton children answered the questions about asthma at age 5 years. Finally, 552 mother-child pairs with phthalate metabolite measurements and information about asthma, eczema and rhinitis were available for inclusion. The participants did not differ from the rest of OCC, however, a smaller proportion of the included mothers smoked during pregnancy (data not shown). The included mothers were on average 31 years old when giving birth and 58% were nullipara (Table 2).

We found that 22.3% of the children had experienced wheeze. Combining self-reported and doctor-diagnosed asthma, the prevalence of asthma was 7.4%. In total, 11.7% had self-reported eczema, whereas 9.2% reported rhinitis (Table 1). Furthermore, 3.3% of the children had both rhinitis and one of the eczema outcomes and 1.7% had rhinitis and one asthma outcome. Only 4 children (0.8%) had an asthma outcome and an eczema outcome.

**Table 1:** Prevalence of allergic outcomes

Outcome	Prevalence
Wheeze within the last 2 years	22.3%
Self-reported asthma	4.7%
Doctor-diagnosed asthma	5.1%
Use of medicine against asthma/cold	13.8%
Self-reported eczema	11.7%
Doctor-diagnosed eczema	6.6%
Medicine against eczema	6.2%
Self-reported rhinitis	9.2%
Rhinitis and eczema	3.3%
Rhinitis and asthma	1.7%
Asthma and eczema	0.8%

Mothers of children with doctor diagnosed asthma had higher BMI and more often had pets. Preterm children wheezed more than children born at term, and children with a birth weight less than 2500 g more often had self-reported asthma compared to children with higher birth weight. Significantly more boys were doctor-diagnosed with asthma and had self-reported rhinitis. The prevalence of wheeze, self-reported asthma, doctor diagnosed asthma and self-reported rhinitis were higher between children with a family history of asthma compared to children without a family history of asthma. Maternal age and parity showed no significant association with asthma, rhinitis or eczema in our study (Table 2).

**Table 2:** Characteristics (percent distribution) of the mother-child pairs according to allergic outcomes

Characteristics	n (%) 552 (100)	Wheeze within the last 2 years (*n* = 552)		Self-reported asthma(*n* = 535)		Doctor-diagnosed asthma (*n* = 530)		Self-reported rhinitis (*n* = 532)		Self-reported eczema (*n* = 487)	
		Yes *n* = 123	No *n* = 429	Yes *n* = 25	No *n* = 510	Yes *n* = 27	No *n* = 503	Yes *n* = 49	No *n* = 483	Yes (57)	No (430)
Sex											
Boy	278 (50.4)	54.5	49.2	48.0	51.2	70.4	49.5	67.3	48.2	43.9	52.3
Girl	274 (49.6)	45.5	50.8	52.0	48.8	29.6*	50.5*	32.7*	51.8*	56.1	47.7
Birth weight (grams)											
< 2500	13 (2.4)	4.1	1.9	8.0*	2.2*	7.4	2.2	4.1	2.3	0.0	2.6
2500–4000	435 (78.8)	74.8	80.0	56.0	80.0	66.7	79.1	71.4	79.9	73.7	80.2
> 4000	104 (18.8)	21.1	18.2	36.0*	17.8*	25.9	18.7	24.5	17.8	26.3	17.2
Preterm birth (<week 37)											
Yes	24 (4.3)	8.9*	3.0*	8.0	4.1	7.4	4.2	6.1	4.1	1.8	4.7
No	528 (95.7)	91.1	97.0	92.0	95.9	92.6	95.8	93.9	95.9	98.2	95.3
Maternal age at delivery (years)											
< 25	50 (9.1)	13.0	7.9	8.0	9.2	11.1	8.9	6.1	9.5	15.8	9.3
25–29	193 (35.0)	35.8	34.7	40.0	34.7	29.6	35.2	36.7	34.6	26.3	36.3
30–34	210 (38.0)	33.3	39.4	32.0	38.4	37.0	38.4	36.7	37.9	36.8	37.4
≥ 35	99 (17.9)	17.9	17.9	20.0	17.6	22.2	17.5	20.4	18.0	21.1	17.0
BMI (kg/m^2^)											
< 20	59 (10.7)	10.6*	10.7*	0.0	11.2	11.1*	10.7*	12.2	10.1	14.0	10.9
20–25	286 (51.8)	42.3	54.5	48.0	52.4	22.2	53.7	38.8	52.6	38.6	53.7
≥ 25	207 (37.5)	47.2*	34.7*	52.0	36.5	66.7*	35.6*	49.0	37.3	47.4	35.3
Parity											
Nulliparous	321 (58.2)	65.0	56.2	64.0	57.5	51.9	57.7	61.2	57.8	63.2	58.4
Multiparous	231 (41.8)	35.0	43.8	36.0	42.5	48.1	42.3	38.8	42.2	36.8	41.6
Smoking during pregnancy											
Yes	16 (2.9)	3.3	2.8	4.0	2.9	3.7	3.0	6.1	2.7	1.8	2.8
No	536 (97.1)	97.7	97.2	96.0	97.0	96.3	97.0	93.9	97.3	98.2	97.2
Education level at pregnancy											
High school or less	144 (26.3)	38.3*	23.0*	20.0*	26.5*	33.3	25.7	34.7	25.7	33.3	26.8
High school + 1–4 years	279 (51.0)	41.7	53.6	36.0	51.5	48.1	51.0	51.0	50.0	50.9	51.4
High school + > 4 years	124 (22.7)	20.0*	23.4*	44.0*	22.0*	18.5	23.3	14.3	24.3	15.8	21.8
Breastfeeding (weeks)											
< 4	45 (9.4)	9.1	9.2	13.6*	9.3*	9.1	9.2	13.3*	9.4*	9.8	10.2
4–19	88 (18.4)	27.3	17.7	36.4	16.8	27.3	17.7	33.3	16.8	17.6	18.7
> 19	345 (72.2)	63.6	73.1	50.0*	73.9*	63.6	73.1	53.3*	73.8*	72.5	71.1
Family history of asthma/allergy											
Yes	309 (56.0)	61.8	54.3	68.0	55.4	63.0	55.6	72.9*	54.9*	69.6	56.5
No	243 (44.0)	38.2	45.7	32.0	44.6	37.0	44.4	27.1	45.1	30.4	43.5
Family history of asthma											
Yes	115 (20.8)	30.1*	18.2*	48.0*	18.6*	44.4*	18.9*	30.6*	18.8*	26.3	19.5
No	437 (79.2)	69.9	81.8	52.0	81.4	55.6	81.1	69.4	81.2	73.7	80.5
Pets (inside and outside)											
Yes	231 (42.4)	50.0	40.2	52.0*	42.3*	59.3*	41.7*	63.8	43.4	37.0	42.6
No	314 (57.6)	50.0	59.8	48.0	57.7	40.7	58.3	36.2	56.6	63.0	57.4

*p-value < 0.05 when testing for significant differences in distribution between the group with an allergic outcome and the group without (using t-test for continuous and chi2-test for categorical variables)

To examine the levels of phthalate exposure, we measured the urinary phthalate concentrations and calculated the percentage of pregnant women who excreted levels above LOD. Phthalate metabolites were detectable in more than 90% of urine samples, except MBzP, which was detected in 68% of the samples (Table 3). Mothers with high or low BMI excreted significantly higher concentrations of phthalate metabolites than normal weight women (Table 4), otherwise no differences in maternal and child characteristics according to phthalate exposure were found.

**Table 3:** Urinary phthalate metabolite levels [ng/mL] measured in gestational week 28

Diether phthalate	Phthalate metabolite	LOD	% > LOD	Mean	Minimum	Percentile					Maximum
						5th	25th	50th	75th	95th	
DEP	MEP	0.53	99.8	103.8	0.37	2.6	7.3	17.9	55.8	351.5	5380.9
DiBP	MiBP	1.10	99.6	37.1	0.78	3.4	12.3	27.1	47.2	104.1	455.2
DnBP	MnBP	1.43	95.3	18.2	<LOD	1.5	6.0	13.5	24.9	51.7	184.3
	ΣMBP_(i + n)_			55.3	<LOD	4.9	19.1	41.5	74.7	150.3	516.1
BBzP	MBzP	1.14	67.9	4.63			<LOD	2.5	5.8	15.7	71.2
DEHP	MEHP	0.14	90.2	1.7		<LOD	0.46	1.1	2.2	5.5	36.8
	MEHHP	0.91	89.3	6.6		<LOD	2.2	51.0	8.6	18.5	92.7
	MEOHP	0.67	91.5	5.4	<LOD	<LOD	1.8	4.3	7.0	14.6	56.6
	MECPP	0.55	96.9	6.4	<LOD	0.73	2.4	5.2	8.5	15.6	95.7
	ΣDEHP_m_			26.5	<LOD	1.5	9.4	21.4	34.6	69.0	305.0
DiNP	MiNP	0.61	11.6	1.2					<LOD	0.98	135.5
	MHiNP	0.26	91.1	9.4		<LOD	0.78	1.6	3.8	12.6	1433.4
	MOiNP	0.25	83.3	5.0		<LOD	0.45	1.1	2.5	8.9	690.4
	MCiOP	0.11	100.0	13.3	0.15	0.52	1.8	3.7	7.5	23.6	1438.4
	ΣDiNP_m_			38.1	0.22	0.77	4.3	8.5	18.3	58.0	4956.8

Abbreviations: *LOD* Limit of detection; *DEP* Diethyl phthalate; *DiBP* Diisobutyl phthalate; *DnBP* Di-n-butyl phthalate; *BBzP* Benzyl-butyl phthalate; *DEHP* Di-2-ethylhexyl phthalate; *DiNP* Diisononyl phthalate; *MEP* monoethyl phthalate; *MiBP* mono-isobutyl phthalate; *MnBP* mono-n-butyl phthalate; *MBzP* monobenzyl phthalate; *MEHP* mono-2-ethylhexyl phthalate; *MEHHP* mono-2-ethyl-5-hydroxyhexyl phthalate; *MEOHP* mono-2-ethyl-5-oxohexyl phthalate; *MECPP* mono-2-ethyl-5-carboxypentyl phthalate; *MiNP* mono-iso-nonyl phthalate; *MHiNP* mono-hydroxy-iso-nonyl phthalate; *MHiNP* mono-hydroxy-iso-nonyl phthalate; *MCiOP* mono-carboxy-iso-octyl phthalate

**Table 4:** Maternal median (25–75 percentile) osmolality adjusted urinary phthalate metabolite concentrations according to mother/child characteristics

Characteristics	n	MEP Median and 25-75th percentile (ng/ml)	ΣMBP_i + n_ Median and 25-75th percentile (ng/ml)	ΣDEHP_m_ Median and 25-75th percentile (ng/ml)	ΣDiNP_m_ Median and 25-75th percentile (ng/ml)
Sex					
Boy	278	22.2 (9.6–55.0)	44.7 (27.1–71.4)	22.0 (13.9–33.2)	9.9 (5.8–19.7)
Girl	274	19.6 (8.8–51.9)	43.7 (26.3–69.2)	21.8 (12.5–31.9)	8.6 (5.1–15.8)
Birth weight (grams)					
< 2500	13	13.4 (6.8–20.9)	37.7 (30.2–44.1)	20.2 (15.8–22.3)	7.2 (6.3–19.2)
2500–4000	435	22.6 (9.8–51.9)	45.2 (27.2–68.5)	22.3 (13.5–33.0)	9.0 (5.4–17.6)
≥ 4000	104	16.6 (8.0–65.6)	42.5 (25.7–73.5)	20.8 (12.3–30.1)	10.8 (5.1–20.4)
Preterm (<week 37)					
Yes	24	17.8 (9.9–72.5)	40.9 (28.8–70.1)	20.8 (12.0–27.2)	9.5 (5.7–20.7)
No	528	21.4 (9.1–52.4)	44.5 (26.6–70.0)	22.0 (13.5–32.9)	9.2 (5.4–18.3)
Age at birth (year)					
< 25	50	20.6 (8.9–37.7)	44.3 (24.3–70.0)	20.4 (14.7–33.0)	9.3 (4.5–14.3)
25–29	193	19.8 (9.8–47.7)	40.5 (26.1–66.2)	21.2 (11.2–31.3)	9.1 (5.1–19.3)
30–34	210	20.5 (8.9–55.0)	45.0 (26.6–70.4)	21.7 (14.2–32.5)	9.3 (5.7–15.5)
≥ 35	99	25.2 (8.8–94.8)	52.1 (35.7–75.1)	24.0 (13.8–34.7)	9.2 (6.4–19.7)
BMI (kg/m^2^)					
< 20	59	26.1 (10.6–55.0)	49.1 (30.2–72.5)*	22.0 (15.0–29.3)*	7.4 (5.0–19.3)*
20–25	286	19.5 (8.7–46.9)	40.4 (22.4–63.0)	20.4 (11.6–31.0)	8.4 (5.0–14.8)
≥ 25	207	25.4 (9.3–65.4)	49.9 (30.5–75.4)*	24.0 (15.8–39.8)*	11.5 (6.6–20.5)*
Parity					
Nulliparous	321	20.9 (10.4–50.0)	44.9 (27.0–69.4)	21.8 (13.4–33.0)	9.5 (5.4–19.2)
Multiparous	231	21.4 (8.6–61.5)	43.9 (26.5–70.1)	22.0 (13.5–32.1)	9.1 (5.2–15.8)
Smoking					
Yes	16	26.2 (7.9–69.2)	48.2 (25.8–76.4)	24.1 (15.7–35.8)	11.4 (4.7–20.4)
No	536	21.0 (9.3–52.5)	44.4 (26.7–69.7)	21.9 (13.5–32.4)	9.2 (5.4–18.3)
Education level years					
Low	144	25.4 (10.6–65.6)	48.5 (29.7–75.6)	24.0 (13.5–39.0)	10.2 (6.0–20.7)
Intermediate	279	19.8 (8.8–55.8)	43.0 (25.3–66.9)	21.4 (13.5–31.0)	9.0 (5.5–16.8)
High	124	19.8 (9.1–39.6)	45.8 (29.4–73.2)	21.7 (12.9–31.6)	8.5 (4.9–19.1)
Breastfeeding					
< 4 weeks	45	17.0 (9.0–53.2)	48.1 (36.1–75.2)	24.8 (13.7–33.4)	10.3 (7.0–21.2)
4–19 weeks	88	30.3 (14.2–91.4)	49.9 (31.5–67.5)	20.7 (15.2–32.8)	12.8 (7.0–19.9)
> 19 weeks	419	20.4 (9.3–49.2)	43.3 (25.7–70.1)	21.8 (13.1–32.1)	8.6 (5.1–17.2)
Family history of asthma/allergy					
Yes	309	19.5 (8.4–49.9)	44.4 (27.2–68.5)	21.6 (13.0–32.4)	9.7 (5.3–20.3)
No	239	23.2 (10.9–56.9)	44.2 (26.0–71.1)	22.3 (13.6–32.8)	8.4 (5.4–14.8)
Family history of asthma					
Yes	115	22.2 (8.1–54.1)	41.2 (26.7–68.5)	21.2 (13.0–32.4)	9.0 (5.1–20.0)
No	437	20.9 (10.2–52.5)	45.2 (26.6–70.0)	22.1 (13.5–32.5)	9.2 (5.6–18.0)

Abbreviations: *MEP* monoethyl phthalate; *ΣMBP*_*i + n*_ Monobutyl phthalate (i + n); *ΣDiNP*_*m*_ sum of Di-isonyl phthalate metabolites; *ΣDEHP*_*m*_ sum of Di-2-ethylhexyl phthalate metabolites

*p < 0.05 using Mann-Whitney test for difference between characteristics with 2 parameters, Kruskal-Wallis test for characteristics with more than 2 parameters

We then compared urinary phthalate concentrations in mothers of children with an allergic outcome to urinary concentrations in mothers of children without the outcome to detect differences. No significant differences in phthalate concentrations were found between children with and without asthma or eczema. Compared to children without rhinitis, children with self-reported rhinitis had been exposed to higher levels of DiNP, DEHP and DBP prenatally (only significant for DiNP). In contrast, mothers of children with asthma, rhinitis or eczema tended to have lower concentrations of MEP compared to mothers of non-allergic children (Table 5). No differences in urinary phthalate concentrations between children with two allergic outcomes compared to children with one or no allergic outcomes were found (data not shown).

**Table 5:** Maternal median (25–75 percentile) urinary phthalate metabolite concentrations among offspring with or without allergic outcomes

Outcome	n	MEP Median and 25–75 (ng/mL)	ΣMBP_i + n_ Median and 25–75 (ng/mL)	ΣDEHP_m_ Median and 25–75 (ng/mL)	ΣDiNP_m_ Median and 25–75 (ng/mL)
Wheeze within the last 2 years					
Yes	123	21.9 (9.6–56.9)	39.7 (25.4–71.5)	22.3 (12.2–33.8)	9.4 (5.4–15.2)
No	429	20.3 (9.0–51.9)	45.2 (27.0–70.0)	21.8 (13.7–32.3)	9.2 (5.4–19.1)
Self-reported asthma					
Yes	25	19.3 (12.7–67.2)	41.9 (30.2–71.5)	22.3 (13.9–33.8)	10.7 (5.1–22.3)
No	510	21.4 (9.1–52.5)	44.5 (26.6–68.5)	21.8 (13.5–32.4)	9.2 (5.4–18.3)
Doctor-diagnosed asthma					
Yes	27	16.4 (6.8–55.5)	36.9 (18.3–74.2)	23.1 (9.3–31.9)	10.3 (4.0–22.3)
No	503	21.8 (9.3–52.5)	44.8 (27.1–68.5)	21.9 (13.5–32.8)	9.3 (5.4–18.4)
Use of medicine for asthma/cold					
Yes	76	21.8 (10.3–66.3)	38.7 (30.0–64.2)	20.6 (9.6–32.6)	8.6 (5.0–14.1)
No	476	20.7 (9.1–52.0)	45.2 (26.3–71.3)	22.0 (13.5–32.4)	9.4 (5.5–19.2)
Self-reported eczema					
Yes	57	16.0 (8.9–35.4)	43.5 (24.8–63.3)	9.6 (5.1–20.7)	21.5 (13.4–31.3)
No	430	22.2 (10.2–55.5)	44.5 (27.2–68.5)	9.4 (5.5–19.0)	22.2 (13.7–33.4)
Doctor-diagnosed eczema					
Yes	32	14.4 (8.6–35.2)	43.1 (19.9–60.4)	9.6 (4.7–22.7)	20.3 (12.1–31.8)
No	455	22.2 (9.7–55.0)	44.6 (27.2–69.2)	9.4 (5.5–18.8)	22.3 (13.8–33.2)
Use of medicine against eczema					
Yes	24	18.2 (8.57–38.8)	40.0 (23.6–62.5)	11.0 (4.7–36.1)	21.4 (13.4–32.7)
No	366	23.7 (10.6–55.6)	44.3 (27.9–68.5)	9.7 (5.7–18.0)	22.1 (14.0–33.4)
Self-reported rhinitis					
Yes	49	17.8 (9.1–43.1)	49.9 (32.0–81.9)	25.2 (16.7–32.1)	11.2^a^ (7.1–21.5)
No	483	21.2 (8.9–55.0)	44.1 (26.6–68.5)	21.8 (13.5–33.0)	9.0 (5.2–18.0)

Abbreviations: *MEP* monoethyl phthalate; *ΣMBP*_*i + n*_ Monobutyl phthalate (i + n); *ΣDiNP*_*m*_ sum of Di-isonyl phthalate metabolites; *ΣDEHP*_*m*_ sum of Di-2-ethylhexyl phthalate metabolites

^a^Significant difference in urinary phthalate concentrations between the group that answered “yes” and the group that answered “no” (using Mann-Whitney test)

Finally, we examined the association between urinary phthalate concentrations and ORs of allergic outcomes to study patterns or possibly significant associations in the study population. In adjusted logistic regression no clear associations were found, however, most ORs for asthma outcomes were below one. Maternal MEP concentration was associated with reduced odds of self-reported (OR = 0.81, 95% CI: 0.68,0.96) and doctor diagnosed eczema (OR = 0.82, 95% CI: 0.66,1.01) in the offspring. A doubling in ΣDiNP_m_ tended to increase the odds of using medication to treat eczema (OR = 1.24, 1.00,1.55). A doubling in ΣDiNP_m_ and ΣDEHP_m_ concentration increased the odds of having self-reported rhinitis, although not statistically significant (OR = 1.15, 95% CI: 0.97,1.36 and OR = 1.21, 95% CI: 0.93,1.58, respectively) (Table 6).

**Table 6:** Adjusted odds ratios and 95% confidence interval for allergic outcomes

Outcome	MEP OR and 95% CI	ΣMBP OR and 95% CI	ΣDEHP_m_ OR and 95% CI	ΣDiNP_m_ OR and 95% CI
Wheeze within the last 2 years	1.00 (0.90,1.11)	0.86 (0.72,1.04)	0.91 (0.78,1.07)	0.89 (0.78,1.02)
Self-reported asthma	1.02 (0.82,1.27)	0.95 (0.67,1.36)	0.98 (0.72,1.33)	0.99 (0.77,1.28)
Doctor-diagnosed asthma	0.91 (0.73,1.12)	0.80 (0.57,1.13)	0.91 (0.68,1.21)	0.94 (0.73,1.21)
Use of medicine against asthma/cold	0.99 (0.88,1.13)	0.86 (0.70,1.08)	0.87 (0.72,1.04)	0.85 (0.72,1.00)
Self-reported eczema	0.81*(0.68,0.96)	0.90 (0.70,1.15)	1.00 (0.80,1.26)	1.03 (0.87,1.22)
Doctor-diagnosed eczema	0.82 (0.66,1.01)	0.79 (0.55,1.10)	0.94 (0.71,1.25)	1.10 (0.90,1.36)
Medicine against eczema	0.88 (0.70,1.10)	0.84 (0.58,1.23)	1.02 (0.72,1.43)	1.24 (1.00,1.55)
Self-reported rhinitis	0.91 (0.75,1.04)	1.11 (0.84,1.46)	1.21 (0.93,1.58)	1.15 (0.97,1.36)

Abbreviations: *OR* odds ratio; *95% CI* 95% confidence interval; *MEP* monoethyl phthalate; *ΣMBP* Monobutyl phthalate (i + n), *ΣDiNP*_*m*_ sum of Di-isonyl phthalate metabolites; *ΣDEHP*_*m*_ sum of Di-2-ethylhexyl phthalate metabolites

Note: ORs were adjusted for maternal age, maternal education, parity and family history of asthma/allergy. * = *p* < 0.05

## Discussion

In this prospective cohort with lower phthalate exposure than similar previously reported cohorts [7, 8, 12, 18, 19] we found no consistent associations between prenatal phthalate exposure and allergic outcomes in the offspring aged 5 years. Interestingly, prenatal DiNP and DEHP exposure tended to increase the risk of rhinitis, although not statistically significant. DEP exposure, measured as urinary MEP concentration, decreased the odds of eczema, while DiNP exposure tended to increase it. The reduction in eczema among DEP exposed may be explained by avoidance behavior, as mothers in families with a history of asthma/allergy had lower MEP-concentrations than mothers in families without asthma/allergy, indicating that they may use less creams and cosmetics containing DEP [26]. In contrast, DiNP is difficult to avoid, as it is found in flooring, building material etc. [4], and we found no differences in DiNP metabolite concentrations between mothers in predisposed and non-disposed families. However, mothers from families with a history of asthma/allergy did not have significantly lower MEP-concentrations compared to mothers from families without, so other parameters may play a role in the association.

Nine cohort studies [7, 8, 10, 14–19] examined the association between prenatal phthalate exposure and wheeze and asthma. Some [8, 10, 14, 16, 17, 19] found association between maternal MnBP and DEHP metabolites and increased risk of wheeze or asthma in children aged 5–11 years. However, in one study [7] a higher DEP exposure decreased the risk of wheeze in girls aged 6–7 years. One study [15] found no associations between MEP, MnBP or MiBP and asthma. We did not find associations between phthalate exposure and asthma, probably because our mothers were less exposed and the diagnosis of asthma is difficult to verify before the children can collaborate [2].

Ten cohort studies [5, 7, 9, 11–16, 19] examined the association between prenatal phthalate exposure and eczema. Two studies [14, 16] measured exposure in serum and detected an association between high concentration of MEHP and DiNP metabolites and decreased risk of eczema. Three studies [11–13] found that higher urinary concentrations of MBzP, MEP, MiBP and ΣDEHP_m_ was associated with increased odds of eczema in 2–5 years old children. One study [5] suggested that higher urinary MBzP concentration increased the risk of eczema in 2-year old but not 5-year old children. Among Polish 2-year-olds, neither MEP, MBP, ΣDiNP_m_ nor ΣDEHP_m_ were associated with eczema [9]. In our cohort DiNP exposure tended to increase the risk of using medicine against eczema, despite low exposure. Three studies [7, 15, 19] examined MEP and MBP and eczema in 7-year-olds, and found no associations possibly because eczema often resolves at child age 6–7 years [27].

One study [15] found no association between maternal urinary concentrations of MEP, MnBP and MiBP and rhinitis in the offspring aged 3 years. To our knowledge, we are the first to report association between prenatal DiNP and DEHP exposure and rhinitis in 5-year-old children, but our findings need confirmation in larger cohorts of older children.

Phthalates are hypothesized to interfere with the immune and respiratory systems and promote the hyperresponsiveness seen in atopic diseases [28]. In vitro, DEHP promotes Th2 polarization and enhances interleukin [28, 29], which causes IgE-production, smooth muscle contractility and mucus production [30]. Furthermore, DEHP interacts with the nuclear hormone receptor family peroxisome proliferator-activated receptors, affecting alveolar maturation and reducing surfactant production, altering lung mechanics [31]. Animal studies indicate that early life exposure to DEHP results in proinflammatory immune responses [32].

Our study was large, population-based and prospective establishing temporal relationship between phthalate exposure and allergic disease. However, our children are most likely healthier than the general population as the prevalence of asthma was 7.4%, whereas it is 12% based on hospital contacts and disease-specific prescribed medications in a large study of Danish 5-year-old children [1]. The participation rate in OCC was 43%, and participating mothers were older and more often non-smokers compared to not participating mothers [20]. As young maternal age and smoking are well known risk factors for asthma this may explain the lower prevalence of asthma in our study [33].

Information on allergy symptoms was obtained from questionnaires, which may be less accurate than the standard diagnostic criteria used by doctors. However, we used a modified version of the validated ISAAC questionnaire and misclassification of symptoms is unlikely to be related to phthalate exposure. Furthermore, more urine samples collected throughout pregnancy would have minimized the risk of misclassification due to temporal exposure variability. Given that the most vulnerable time for exposure to endocrine disrupting chemicals during pregnancy has not yet been determined, we may have misclassified exposures by measuring concentrations in the third trimester. Such nondifferental misclassifications can possibly underestimate outcomes related to phthalate exposure, which may explain our odds ratios close to 1. We detected a wide variation of phthalate concentrations in the urine samples (Table 3). The wide variation is normal for human-biomonitoring studies of phthalates, and several longitudinal studies show that the intraindividual variation of the phthalates are most often lower than the interindividual variation [6, 34].

Although our cohort was relatively large, few children reported allergic symptoms, which made it difficult to adjust for more covariates without losing statistical power. Thus, we did not account for the contribution of childhood exposures, although studies have found association between postnatal phthalate exposure and development of asthma and allergy [10, 35]. Likewise, we did not adjust for exposure to other phthalates or other endocrine disrupting chemicals. Our correlation analysis showed moderate to high correlation between phthalate metabolites and distinguishing the effect of exposure to one phthalate from another phthalate is difficult. In addition, we tested many potential associations and our significant result may be due to chance.

## Conclusions

In this low exposed prospective mother-child cohort we found no consistent association between maternal phthalate exposure and asthma and eczema in the offspring aged 5-years. Prenatal exposure to DiNP and DEHP was associated with rhinitis, although not significantly, possibly due to a relatively small number of 5-year-old children with rhinitis. Given the widespread use of phthalates, the possible association with rhinitis is of public health concern. It is therefore important to follow up the children with allergy testing, lung function assessment and phthalate exposure measurements, as the prevalence and diagnostic accuracy of allergic diseases will increase with age.

## Data Availability

The dataset supporting the conclusions of this article is available upon request.

## Abbreviations

BMI: Body Mass Index
DEHP: Di-2-ethylhexyl phthalate
DiNP: Diisononyl phthalate
GA: Gestational age
ISAAC: International Study of Asthma and Allergies in Childhood
Ln: Natural logarithm
LOD: Limit of detection
MBP: Mono-butyl phthalate
MBzP: Mono-benzyl phthalate
MCiOP: Mono-carboxy-iso-octyl phthalate
MECPP: Mono-2-ethyl-5-carboxypentyl phthalate
MEHHP: Mono-2-ethyl-5-hydroxyhexyl phthalate
MEHP: Mono-2-ethylhexyl phthalate
MEOHP: Mono-2-ethyl-5-oxohexyl phthalate
MEP: Monoethyl phthalate
MHiNP: Mono-hydroxy-iso-nonyl phthalate
MiBP: Mono-iso-butyl phthalate
MiNP: Mono-iso-nonyl phthalate
MnBP: Mono-*n*-butyl phthalate
MOiNP: Mono-oxo-iso-nonyl phthalate
OCC: Odense Child Cohort
